# Prevalence of diagnosable depression in patients awaiting orthopaedic specialist consultation: a cross-sectional analysis

**DOI:** 10.1186/s12891-023-06688-0

**Published:** 2023-07-22

**Authors:** Rhiannon K. Patten, Alev Asilioglu, Itamar Levinger, Alexander Tacey, Michaela Pascoe, Phong Tran, Michael J. McKenna, Catherine M. Said, Natali Coric, Mary De Gori, Rebecca Lane, Vasso Apostolopoulos, Mary N. Woessner, Alexandra Parker

**Affiliations:** 1 Institute for Health and Sport, Victoria University, Melbourne, Australia; 2 Department of Orthopaedic Surgery, Western Health, Melbourne, Australia; 3 Australian Institute for Musculoskeletal Science (AIMSS), Victoria University, University of Melbourne and Western Health, Melbourne, Australia; 4 Physiotherapy, Melbourne School of Health Sciences, University of Melbourne, Melbourne, Australia; 5 Physiotherapy, Western Health, Melbourne, Australia

**Keywords:** Osteoarthritis, Depression, Pain, Waitlist, Orthopaedic, Specialist care, Musculoskeletal

## Abstract

**Background:**

Musculoskeletal conditions, including osteoarthritis (OA), are a leading cause of disability and chronic pain, and are associated with high rates of comorbid depression. However, signs of depression are often masked by pain. The aim of this study was to determine the prevalence and severity of depression and pain in individuals awaiting specialist orthopaedic consultation. A secondary objective was to determine the relationship between pain and depression, irrespective of demographic factors and clinical diagnosis.

**Methods:**

Cross-sectional analysis of individuals awaiting orthopaedic consultation at a public hospital in Melbourne, Australia. Relevant data were extracted from medical records and questionnaires. Descriptive statistics were used to summarise participant characteristics. The patient health questionnaire (PHQ-9) was used to assess depression and a numerical rating scale (NRS) was used to assess pain severity. Multiple linear regression analyses were used to establish the relationship between pain and depression.

**Results:**

Nine hundred and eighty-six adults (mean ± standard deviation, age = 54.1 ± 15.7 years, 53.2% women) participated in the study. OA was present in 56% of the population and 34% of the entire population had moderate depression or greater, 19% of which met the criteria for major depressive disorder. Moderate-to-severe pain was present in 79% of individuals with OA and 55% of individuals with other musculoskeletal complaints. Pain was significantly associated with depression scores (β = 0.84, adjusted R^2^ = 0.13, P < 0.001), and this relationship remained significant after accounting for gender, age, education and employment status, OA status, number of joints affected and waiting time (β = 0.91, adjusted R^2^ = 0.19, P < 0.001).

**Conclusions:**

Depression affects one-third of individuals on an orthopaedic waitlist. A strong link between pain and depression in patients awaiting specialist orthopaedic consultation exists, indicating a need for an integrated approach in addressing pain management and depression to manage this complex and comorbid presentation.

## Introduction

Approximately one third of Australians report having arthritis or other musculoskeletal conditions [1]. Musculoskeletal conditions can have a profound impact on a person’s health, causing chronic pain and functional limitations leading to reductions in physical and mental wellbeing [2]. Osteoarthritis (OA) is one of the most prevalent musculoskeletal conditions, affecting approximately 2.2 million Australians [3] and 250 million people worldwide [4]. OA of the knee and hip is the third most prevalent musculoskeletal disorder and is ranked as the 11th highest contributor to global disability [5]. The high prevalence of musculoskeletal conditions within the community generates a significant demand for specialised care, often exceeding the number of available appointments, necessitating the use of a waitlist. The Australian public hospital system is experiencing long delays, made worse by the COVID-19 pandemic resulting in extensive waiting periods for specialist orthopaedic consults [6]. This delay in treatment can result in a worsening of symptoms, more advanced pain, and can lead to a deterioration in mental health and quality of life [7, 8].

Chronic pain is often the catalyst that leads individuals with musculoskeletal problems to seek medical care [9]. Chronic pain and depressed mood often coexist, with depression being a common comorbidity of many chronic diseases including OA [10, 11]. There is a larger burden when pain and depression coincide and a pressing need to explore and address this co-occurrence. In individuals with OA, a recent meta-analysis showed a moderate positive correlation between pain severity and symptomology [12], however, whether this relationship exists in a broader range of patients with orthopaedic complaints is yet to be determined.

Individuals affected by OA are nearly three times more likely to report very severe pain, and two times as likely to report high levels of psychological distress compared to those without OA [3]. However, most of the current research is in a general population and little is known about the impact in a public health setting. Given the extensive wait times, this information could assist in determining the most appropriate care plan whilst patients are awaiting consultation. Therefore, the objective of this study was to assess the prevalence and severity of pain and depression in patients with and without OA who are on an orthopaedic waitlist at a public hospital in Melbourne, Australia. The secondary aim was to determine the association between pain and depression in this cohort.

## Methods

### Participants

Participants were recruited from the orthopaedic outpatient waitlist at Western Health, a metropolitan public tertiary teaching hospital in Melbourne, Australia. Recruitment began August 2021 and is ongoing. All patients placed on the waitlist between the 1st of January 2018 and the 1st of June 2022 who were 18 years of age or older were invited to participate. Participants were excluded if they had a cognitive impairment, or if they had a specialist appointment scheduled.

### Procedures

This study is part of a larger research project which is collecting longitudinal data examining the health and wellbeing of individuals on the orthopaedic waitlist in order to determine their needs and develop effective interventions. The study was approved by Melbourne Health Human Research Ethics Committee (2021.055) and site specific approval was granted by Western Health. Eligible patients were contacted via telephone call. Three attempts were made to contact patients. Those unable to be contacted after three attempts were recorded as non-responders. Interested patients were sent a survey package via email or mail, or completed over the phone. For patients preferring an electronic survey, a link to a secure website (REDCap) where patients could complete the survey was provided. For patients that preferred hard-copy, surveys were mailed out with an addressed, prepaid envelope. If patients elected to complete the survey over the phone, the survey questions and response options were read to them and the response was recorded on their behalf. Informed consent was obtained prior to data collection.

### Measures

For all patients on the waitlist, data were extracted from their medical records and entered into a secure online database (REDCap). Extracted data included date of birth, postcode, age, date that the GP referral was triaged by the hospital, number of joints affected, location/s of affected joint/s and whether osteoarthritis is present or suspected. Participants completed a survey comprising a range of self-report questionnaires including demographics, medical history and health status, pain and psychological wellbeing. The questionnaires were in English, however, if patients were unable to understand or read English well, their next of kin was contacted for assistance.

Patients were listed as having OA if it was listed on their referral, or if they met the three clinical criteria; (1) over the age of 45 years; (2) has activity-related joint pain; and (3) has no morning joint-related stiffness, or morning stiffness that lasts no longer than 30 min [13], determined within the survey. If they did not meet these criteria, they were listed as not having OA.

### Questionnaires

The Patient Health Questionnaire (PHQ-9) was used to assess patients’ level of depression over the previous two weeks [14]. The PHQ-9 is a self-report measure consisting of nine-items matching the Diagnostic and Statistical Manual of Mental Disorders criteria for major depression. Questions are rated on a four-point Likert scale ranging from 0 (“not at all”) to 3 (“nearly every day”). Scores are summed together, and can range from 0 to 27. Scores of 5, 10, 15, and 20 represent mild, moderate, moderately severe and severe depression, respectively. Consistent with clinical usage of the PHQ-9, a score of ≥ 10 was considered a positive screen for depression [14]. Participants were suggested to have major depression if 5 or more of the 9 depressive symptom criteria were present at least “more than half the days” in the past two weeks, and 1 of the symptoms is depressed mood or anhedonia. The question regarding suicidal ideation counted if present at all [14].

Pain intensity was self-rated by participants using the numeric rating scale (NRS). The NRS is an 11-point numerical scale ranging from 0 (no pain) to 10 (worst possible pain) [15]. Various cut-off points have been used to analyse this outcome. Here we use scores of 0, 1, 5 and 8 to represent none, mild, moderate and severe pain, respectively [16].

### Data analysis

All statistical analyses were conducted using R version 4.1.2 (R Core Team, Vienna, Austria) in RStudio (RStudio Team, Boston, MA). Descriptive statistics such as mean and standard deviations (SD), and percentages were used to characterise the sample. Patterns of missing data were examined using the naniar package [17] and Little’s multivariate test statistic was used to assess if data were missing completely at random (MCAR) which included all variables. Data was determined to be MCAR (Little’s MCAR x^2^ (57) = 56.0, P = 0.513), therefore, missing data were deleted pairwise. The Kolmogorov–Smirnov test was used to assess normal distribution of data. Data were deemed to be normally distributed with the exception of wait time and a square root transformation was performed for analysis. To test differences amongst the OA and non-OA subgroups for the total score of the survey items, t-test were used. Chi-squared test of independence was used to test for differences between categorical variables for the OA and non-OA subgroups. A simple linear regression was conducted to determine the relationship between pain and depression scores. Multiple linear regression analyses were used to identify whether pain and demographic characteristics were associated with depression scores (model 1) and lastly, to determine whether pain, demographic characteristics and joint specific factors were associated with depression scores (model 2). Collinearity statistics were used to evaluate the variation inflation factor (VIF). The VIF values were less than 3 indicating no problematic multi-collinearity [18]. Statistical significance was considered when the P value was < 0.05.

## Results

**Fig. 1:**
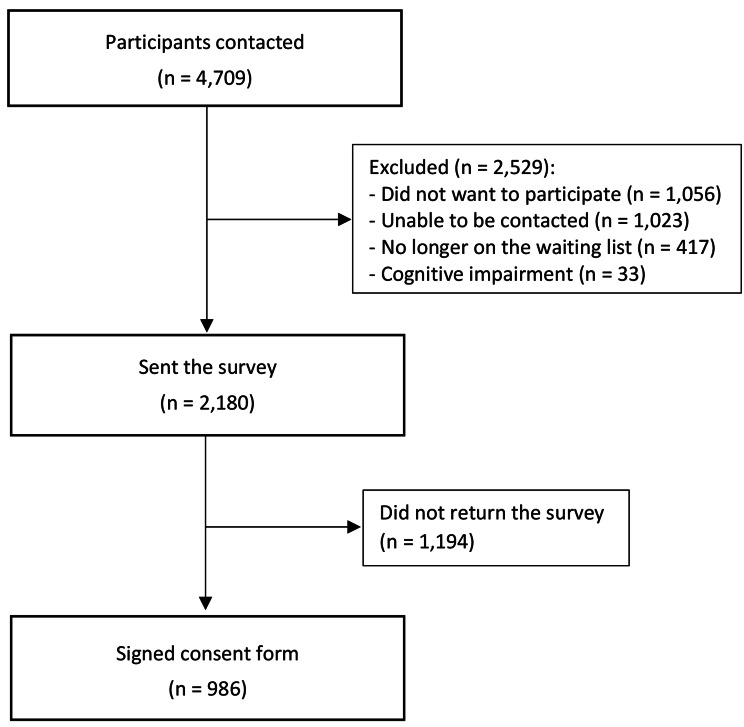
Participant flow diagram

Of the 4,709 patients contacted, 46.3% agreed to participate (n = 2,180), of which 45.2% (n = 986) signed consent. Of the patients contacted, 22.4% refused participation, 21.7% did not respond and 9.6% were deemed ineligible. Participant flow is detailed in Fig. 1.

**Figure legends**.

Demographic characteristics are presented in Table 1. The mean age of the waitlist population was 54.1 ± 15.7 SD. OA was present in 56.0% of the population. The group with OA were older than those without OA (62.6 ± 10.7 vs. 43.3 ± 14.4, P < 0.001). The median wait time at the time of survey completion was 588 days. Just over half of the population identified as women (53.2%). Across both groups, 21.6% of patients had bilateral joints affected, and 3.8% of patients had more than one joint affected (e.g. hip and knee). The knee was the most commonly affected joint across the entire population (40.7%), and accounted for almost half of the OA referrals (47.4%). 70.2% of patients without OA had paid employment, whereas less than half of the OA population had paid employment. Of individuals that were not working, 65.0% of those with OA were retired and 38.8% of those without OA were retired.

**Table 1:** Participant characteristics for the combined waitlist population and sub-groups by OA status

Outcome	OA (n = 552)	Non-OA (n = 434)	Total (n = 986)
Age (years) – mean ± SD	62.6 ± 10.7	43.3 ± 14.4	54.1 ± 15.7
Wait time (days) – median (IQR)	577 (200–786)	596 (195–811)	588 (199–799)
Joint affected – n (%)	n = 745	n = 512	n = 1,257
Knee	353 (47.4%)	158 (30.9%)	511 (40.7%)
Hip	122 (16.4%)	91 (17.8%)	213 (16.9%)
Foot	104 (14.0%)	104 (20.3%)	208 (16.5%)
Shoulder	97 (13.0%)	71 (13.9%)	168 (13.4%)
Ankle	36 (4.8%)	42 (8.2%)	78 (6.2%)
Wrist	11 (1.5%)	15 (2.9%)	32 (2.5%)
Elbow	14 (1.9%)	18 (3.5%)	26 (2.1%)
Hand	4 (0.5%)	3 (0.6%)	7 (0.6%)
Other	4 (0.5%)	10 (2.0%)	14 (1.1%)
Gender – n (%)			
Women	302 (54.7%)	223 (51.4)	525 (53.2%)
Men	249 (45.1%)	208 (47.9%)	457 (46.3%)
Non-binary	1 (0.2%)	3 (0.7%)	4 (0.4%)
Employment – n (%)			
Employed	198 (35.9%)	304 (70.2%)	502 (51.0%)
Not working	354 (64.1%)	129 (29.8%)	483 (49.0%)
Highest education – n (%)			
Did not complete secondary school	256 (46.7%)	101 (23.3%)	357 (36.4%)
Completed secondary school	82 (15.0%)	75 (17.3%)	157 (16.0%)
Certificate or diploma	145 (26.5%)	132 (30.5%)	277 (28.2%)
Undergraduate degree	42 (7.7%)	75 (17.3%)	117 (11.9%)
Postgraduate degree	23 (4.2%)	50 (11.5%)	73 (7.4%)
Country of birth – n (%)			
Australia	295 (53.5%)	267 (61.5%)	562 (57.1%)
Country other than Australia	256 (46.5%)	167 (38.5%)	423 (42.9%)

Abbreviations. IQR: interquartile range, OA: osteoarthritis, SD: standard deviation.

Missing data include: employment and country of birth = 1 record, education = 5 records.

### Depression

**Fig. 2:**
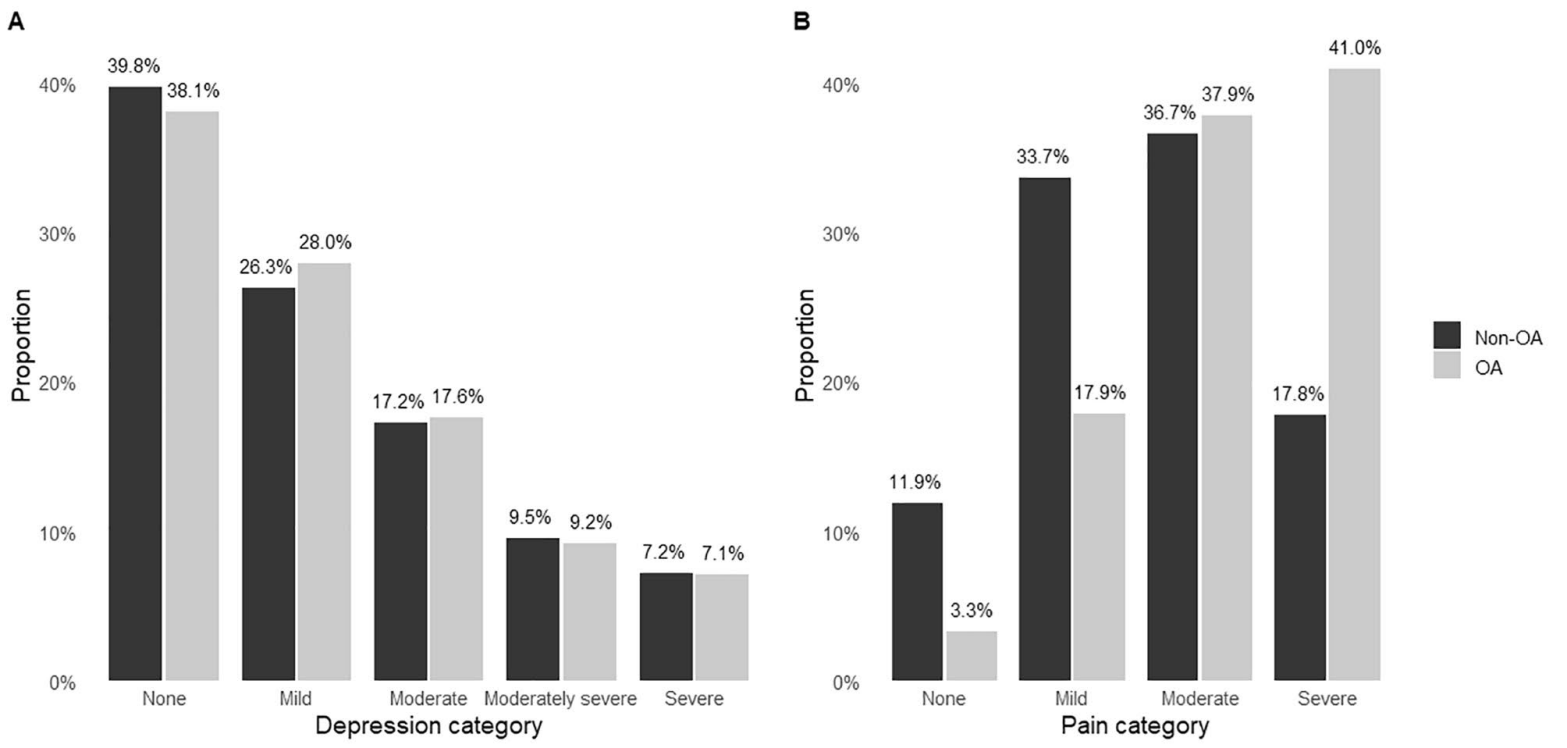
Distribution of individuals in each of the depression categories (A) and pain categories (B) for the OA and non-OA subgroups Note: OA: osteoarthritis. Depression categories according to the patient health questionnaire – 9-items (PHQ-9) and pain categories scored using a numerical rating scale

The mean depression scores were 7.9 ± 6.6 and 7.9 ± 6.7 for the OA and non-OA subgroups, respectively. The mean depression scores did not differ significantly between groups (95% CI: -0.89, 0.87, P = 0.981), nor did the proportion of individuals’ per category (*X*^2^ (4) = 0.440, P = 0.979). The proportion of individuals in each category are presented in Fig. 2. Of note, 33.9% of participants in both groups had moderate or greater depression (score of ≥ 10), and 18.8% of patients with OA and 19.9% of patients without OA met the criteria for major depressive disorder.

### Pain

The mean pain scores were 6.4 ± 2.5 and 4.6 ± 2.8 for the OA and non-OA subgroups, respectively. Scores differed significantly between groups, with higher pain scores in the OA group (95% CI: -2.5, -1.44, P < 0.001), as did the proportion of individuals per category, *X*^2^ (3) = 82.15, P < 0.001. The proportion of individuals in each category is presented in Fig. 2.

### Associations with depression

Initially, univariate linear regression analysis determined that higher pain scores were significantly associated with higher depression scores (β = 0.84 adjusted R^2^ = 0.13, F(1, 884 = 128.40, 95% CI: 0.70, 0.99, P < 0.001)). Following this, multiple linear regression was performed to test whether the addition of age, gender, employment status, education level and country of birth better explained the variance in depression scores (model 1). The overall regression was statistically significant (adjusted R^2^ = 0.19, F(6, 873 = 35.07, P < 0.001)), with higher pain, older age, being a man, and not having employment all being significantly associated with higher depression scores (Table 2). For model 2, the number of joints affected, OA status and wait time were added. The addition of the variables did not further explain depression scores (adjusted R^2^ = 0.19, F(9, 868) = 23.71, P < 0.001)) and none of the additional variables were statistically significantly (Table 2). Of note, pain remains a strong predictor of depression scores after accounting for both demographic and joint characteristics.

**Table 2:** Results of multiple linear regression analyses predicting depression scores

	Model 1			Model 2		
	adjusted R^2^	β	95% CI	adjusted R^2^	β	95% CI
	0.19			0.19		
Pain score		0.90^***^	0.75, 1.06		0.91^***^	0.75, 1.06
**Demographic characteristics**						
Age (years)		-0.12^***^	-0.15, -0.09		-0.12^***^	-0.16, -0.08
Gender: man		-1.00^*^	-1.8, -0.19		-1.00^*^	-1.81, -0.18
Employment status: yes		-2.27^***^	-3.22, -1.31		-2.18^***^	-3.14, -1.21
Education: completed high school		-0.21	-1.11, 0.69		-0.22	-1.12, 0.69
Country of birth: country other than Australia		-0.34	-1.16, 0.49		-0.34	-1.17, 0.49
**Joint characteristics**						
Osteoarthritis: yes					-0.06	-1.10, 0.99
Number of joints affected					0.70	-0.09, 1.49
Wait time (months)					0.01	-0.04, 0.06

^*^P < 0.05, ^**^P < 0.01, ^***^P < 0.001; β: standardised beta coefficient.

## Discussion

We report that a large proportion of individuals on an orthopaedic waitlist have high scores of depression, with 34% meeting the criteria for diagnosable depression and approximately one-fifth of the population meeting the criteria for major depressive disorder. Individuals with and without OA had high rates of moderate-to-severe pain, however, individuals with OA report significantly higher pain scores compared to those without. Amongst all individuals on the waitlist, pain was a significant predictor of depression scores, accounting for 13% of the variation in depression scores.

Approximately 34% of individuals on the orthopaedic waitlist had diagnosable depression according to scores from the PHQ-9, 19% of which met the criteria for major depressive disorder [14]. These results are similar to those reported in individuals with OA [19] but are substantially higher than the general population [20]. This is of particular concern as there is accumulating evidence that depression negatively impacts quality of life, work productivity, prognosis, and can result in greater healthcare expenses [21–23]. Depression is suggested to exacerbate the health-related burden of OA, however, it often goes undetected due to pain and physical function being more prominent [24]. If depression is identified or the individual appears at risk of developing depression, it is recommended that general practitioners or physicians refer patients for psychological treatment. In Australia, this would entail practitioners developing either a chronic diseases management plan where patients can access up to five sessions with a psychologist or exercise physiologist [25], or a mental health care plan enabling patients to access up to 20 sessions [26].

Our finding that moderate-to-severe pain was reported in 66% of individuals on the orthopaedic waitlist is problematic given the significant health and economic burden to the individual, and the healthcare system. In Australia in 2018, chronic pain was estimated to cost ~$139 billion, mostly attributed to loss of productivity and reduced quality of life [27]. A national survey of Australian adults reported a prevalence of chronic pain of 19% [28]. The most commonly reported causal conditions were OA (48.1%) and back problems (29.4%) [28]. Unlike many other conditions where the underlying injury typically resolves, OA does not [29]. Although OA affects individuals of all ages, there is a sharp rise in prevalence from the age of 45 years [3]. Given that OA is a leading cause of pain, this suggests that as the Australian population ages, the prevalence of chronic pain induced by OA will also increase [28].

Pain and depression are closely correlated and often co-occur [30]. High rates of pain and depression have immediate implications for a patients’ health, but may also affect long-term surgical outcomes [31]. Furthermore, the presence of pain has been found to negatively affect the recognition and treatment of depressive symptoms [32]. We found that although individuals with OA reported higher levels of pain compared to those without OA, surprisingly, those with OA did not have higher depression scores. Regardless, we found that pain is significantly associated with depression scores in patients on an orthopaedic waitlist. This suggests that treatment options that impact both pain and depression should be integrated into the management of musculoskeletal conditions. Both exercise and pain coping skills training (PCST) have been identified as effective strategies for managing pain and depression [21]. Individuals can participate in these strategies either in person or via a digital platform. Digital self-management interventions have low attrition rates, are low-cost and remove barriers to face-to-face interventions [33, 34]. These strategies could be integrated into a comprehensive care approach for individuals on lengthy waitlists to mitigate the impact of their condition.

Strengths of this study include the large sample size, which included a broad range of individuals from diverse backgrounds and the use of the PHQ-9 which is a validated, reliable measure and is considered the gold standard for determining depression symptom severity. Limitations of this study include the lack of patient response with less than 25% of approached patients choosing to participate, the cross-sectional nature, and that patients were surveyed after varying lengths of time on the wait list. We were also unable to specify and account for reasons for referral in patients that did not have OA. In addition, the questionnaires were only provided in English. Future studies should endeavour to survey patients in their preferred language and from the time that patients are placed on the waitlist.

## Conclusions

In conclusion, a large proportion of individuals on an orthopaedic waitlist are affected by depression. This indicates a need for action in assessing and addressing both the physical and mental health concerns of individuals whilst they are awaiting consultation. It was also noted that pain is significantly associated with depression and we recommend that the two be addressed simultaneously to reduce the risks associated with depression, and reduce the burden on the individual and the healthcare system.

## Data Availability

The datasets used and/or analysed during the current study are available from the corresponding author on reasonable request.

## List of abbreviations

NRS: Numerical rating scale
OA: Osteoarthritis
PHQ-9: Patient health questionnaire – 9-items
